# Therapeutic options for chronic inflammatory demyelinating polyradiculoneuropathy: a systematic review

**DOI:** 10.1186/1471-2377-14-26

**Published:** 2014-02-07

**Authors:** Richard J Bright, Jenny Wilkinson, Brendon J Coventry

**Affiliations:** 1 Faculty of Health Sciences, School of Dentistry, University of Adelaide, Adelaide, Australia; 2 School of Biomedical Sciences, Charles Sturt University, Wagga Wagga, Australia; 3 Faculty of Health Sciences, Immunotherapy Research Laboratory, Royal Adelaide Hospital, Adelaide, Australia

**Keywords:** Chronic inflammatory demyelinating polyneuropathy, Peripheral neuropathy, Anti-myelin associated glycoprotein, Autoimmune diseases, Treatment, Plasmapheresis, IVIg, Corticosteroids

## Abstract

**Background:**

Chronic inflammatory demyelinating polyradiculoneuropathy is a rare acquired immune-mediated progressive or relapsing disorder causing peripheral neuropathic disease of duration more than two months. Many individuals with chronic inflammatory demyelinating polyradiculoneuropathy fail to make a long-term recovery with current treatment regimes. The aim of this study was to prospectively review the literature to determine the effectiveness of therapies for chronic inflammatory demyelinating polyradiculoneuropathy.

**Methods:**

Articles published from January 1990 to December 2012 were searched for studies to treat adults with chronic inflammatory demyelinating polyradiculoneuropathy. Peer-reviewed full-text articles published in English were included.

**Results:**

Nine placebo-controlled double-blinded randomised trials were reviewed to treat subjects with chronic inflammatory demyelinating polyradiculoneuropathy exhibiting various degrees of effectiveness. The most effect treatments were; three randomised controlled trials using intravenous immunoglobulin, a study comparing pulsed dexamethasone and short term prednisolone and rituximab all showed promising results and were well tolerated.

**Conclusion:**

IVIg and corticosteroids remain first line treatments for CIDP. Therapies using monoclonal antibodies, such as Rituximab and Natalizumab offer the most promise for treatment of Chronic inflammatory demyelinating polyradiculoneuropathy however they also need further research, as does the use of stem cell therapy for treating Chronic inflammatory demyelinating polyradiculoneuropathy. Large randomised controlled trials and better patient selection are required to address responsiveness of CIDP patients to conventional treatments to elucidate mechanisms of action and future directions for therapeutic improvement.

## Background

Chronic inflammatory demyelinating polyradiculoneuropathy (CIDP) is an acquired peripheral neuropathy, with both T and B cell involvement [1]. It is the most common peripheral autoimmune demyelinating neuropathy with a prevalence of 1.2 to 7.7 per 100,000 worldwide, with a slight male predominance [2]. The disease involves progressive loss of immunologic tolerance to peripheral nerve components such as myelin, Schwann cell, the axon, and motor or ganglionic neurons [3,4]. There is increasing evidence that activated macrophages, T cells, and auto-antibodies induce an immune attack against peripheral nerve antigens [4]. Complement-fixing immunoglobulin deposits are localised to the myelin sheath surrounding axons and antibodies to various glycolipids and myelin proteins are frequently detected in subjects with CIDP and other autoimmune neuropathies. Activated tissue macrophages comprise the final process in the demyelinating process by invading the lamellae causing focal damage to the myelin sheath [2]. The resulting demyelination affects spinal roots, proximal nerve trunks and major plexi that lead to loss of strength and sensation, which may explain the variability in clinical presentation [4,5]. The common CIDP variants include unifocal or multifocal, pure motor, pure sensory, sensory ataxic and pure distal forms [4]. Relatively little is known about the pathogenesis of CIDP; however there are many theories proposed. The occurrence of CIDP in individuals with melanoma or those who have been administered melanoma vaccine has previously been reported, however this finding is quite rare [6,7]. As numerous carbohydrate epitopes are shared by melanoma cells and myelin molecular mimicry may be a key factor in the initiation of the condition. More commonly, CIDP may develop after bacterial or viral infection particularly viral hepatitis and post vaccination. It has been suggested that viral and bacterial components have antigenic similarities to the body’s own proteins leading to an auto-immune reaction, or alterations in T cell function [8,9].

Currently there are no biomarkers or no clear genetic predisposition, although approximately 20% of sufferers have paraproteins in their serum, including anti-myelin associated glycoprotein (MAG) antibodies and elevated cerebrospinal fluid protein levels [3]. Antibodies to GM1 ganglioside have been reported in 23% of patients with CIDP [10], while other researchers have observed increased frequency of other antibodies directed against peripheral nerve antigens and in HLA antigens [11,12]. CIDP can be described as a spectrum of diseases requiring early recognition to enable optimum treatment management. The disease follows a progressive, monophasic or relapsing remitting course with clinical signs of CIDP being proximal and distal weakness (usually symmetrical), sensory involvement (numbness) and areflexia. Nerve conduction in CIDP patients may exhibit prolonged distal motor latency, slowed conduction velocity, partial conduction block and delayed or absent F-wave [13]. Current treatments for CIDP include immunomodulating, anti-inflammatory and immunosuppressive drugs, and these have varying degrees of effectiveness. The most commonly used treatments for CIDP include; corticosteroids, intravenous immunoglobulin (IVIg) and plasma exchange (PE). CIDP may improve spontaneously without any intervention making it difficult to judge drug efficacy in small clinical trials [14,15]. Newer immunotherapies targeting B cells, T cells, transmigration molecules and signal transduction pathways may have potential for treating CIDP. This systematic review evaluates the safety and efficacy of randomised control trials treating chronic inflammatory demyelinating polyradiculoneuropathy.

## Methods

### Literature search

PubMed, Embase and The Cochrane Neuromuscular Disease Group Trials Specialized Register were searched from January 1990 to December 2012 inclusive for published articles on ‘chronic inflammatory demyelinating polyradiculoneuropathy’ and ‘treatment’. Medical subject heading (MeSH) search terms were used to search PubMed and a keyword search were used if required. Keyword search terms used were; “chronic inflammatory demyelinating polyneuropathy” or “CIDP” or “chronic inflammatory polyneuropathy” or “autoimmune neuropathies” combined with “drug therapy” or “treatment” or “therapy” or “randomised control trial” or “clinical trial”. Included in this study were double blind randomised controlled trials for treating CIDP. All current and emerging treatments for CIDP were included in the study and papers were excluded if the diagnosis of CIDP was considered secondary to an underlying disorder.

### Study selection and participants

When journal articles did not publish the necessary data for the analyses, attempts were made to contact the authors. This study included adult patients of both sexes diagnosed with CIDP according to the criteria for diagnosis; clinically accepted electrodiagnostic criteria, progression of weakness lasting more than eight weeks and increased cerebrospinal fluid protein [16,17]. However there are numerous sets of accepted criteria for the diagnosis of CIDP, with many variables and there is not one uniform set of criteria. Papers were excluded if subjects had another systemic disease, family history of CIDP or drug or toxin exposure known to cause CIDP. No restrictions were set on suitable settings for involvement in this review.

### Assessment of methodological quality

Studies used in the systematic review were assessed for levels of concealment of allocation at randomisation and internal validity to determine if any bias was present. Quality of evidence for each study was graded from very low to high using GRADEprofiler (http://ims.cochrane.org/gradepro). Each paper was assessed for risk of bias, inconsistency, indirectness, imprecision, publication bias, large effect, plausible confounding would change the effect and dose–response gradient. Each item was graded as either; No- negligible, Level 1- Serious or Level 2- Very Serious. GRADEprofiler software utilised these parameters to report a summarised measure of the quality of evidence from; very low, low, moderate to high (Table 1).

**Table 1:** Summary of findings and quality assessment for the qualitative analysis using grade profiler software

**Randomised clinical studies to treat CIDP- summary of findings**						
**Outcomes**	**Intervention and comparison intervention**	Illustrative comparative risks* (95% CI)		**No of participants (studies)**	**Quality of the evidence (GRADE)**	**References**
		**Assumed risk**	**Corresponding risk**			
		**With comparator**	**With intervention**			
Greater than 20% reduction in mean weekly dose of corticosteroids or IVIG						
	Methotrexate/placebo (OR OR 1.38, 95% CI 0.5-3.87 )	**438 per 1000**	**518 per 1000** (280 to 751)	59 (1 study)	⊕⊕⊕⊝ **Moderate**	[18]
Disease progression @ 32 weeks						
	Interferon/placebo	**474 per 1000**	**343 per 1000** (146 to 620)	54 (1 study)	⊕⊕⊕⊝ **Moderate**	[20]
Responders						
	Immunoadsorption/IVIG	**500 per 1000**	**800 per 1000** (231 to 982)	13 (1 study)	⊕⊝⊝⊝ **Very low**	[22]
	IVIG/placebo	**222 per 1000**	**781 per 1000** (275 to 970)	18 (1 study)	⊕⊕⊕⊝ **Moderate**	[24]
	IVIG/placebo	**231 per 1000**	**266 per 1000** (62 to 671)	28 (1 study)	⊕⊕⊝⊝ **Low**	[25]
	IVIG/placebo	**207 per 1000**	**542 per 1000** (344 to 728)	117 (1 study)	⊕⊕⊕⊕ **High**	[27]
	Rituximab/placebo	**77 per 1000**	**520 per 1000** (49 to 958)	26 (1 study)	⊕⊕⊕⊝ **Moderate**	[31]
Adverse treatment related effects						
	Kiovig (10% liquid immunoglobulin)/Gammagard (5% freeze dried immunoglobulin standard)	**692 per 1000**	**714 per 1000** (321 to 929)	27 (1 study)	⊕⊕⊕⊝ **Moderate**	[29]
Remission at 12 months						
	Pulsed high-dose dexamethasone/standard prednisolone treatment	**375 per 1000**	**417 per 1000** (165 to 723)	40 (1 study)	⊕⊕⊝⊝ **Low**	[32]

### Statistical analysis

**Figure 1:**
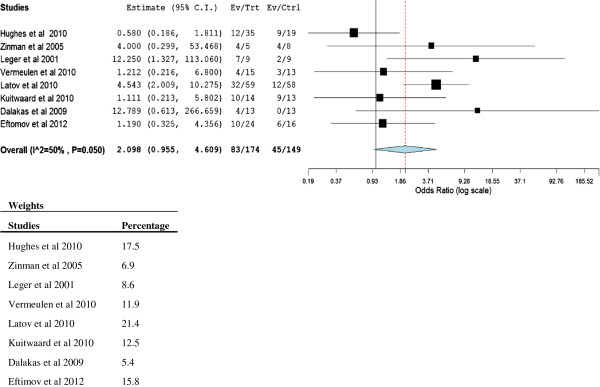
**Meta-analysis of studies to improve recovery of CIDP versus control groups.** Odds ratios are presented with 95 percent confidence intervals. Overall effect is shown using DerSimonian-Laird (DL) binary random- effect model.

For the nine randomised controlled trials in this analysis, the proportion of patients with significant improvement in disability or the proportion that exhibited adverse effects for the treatment were used to calculate the odds ratio and 95% confidence intervals for each study. Assumed and corresponding risks (95% confidence interval) were calculated using GRADEprofiler Version 3.6 software. A summary of findings was also created using GRADEprofiler software. Meta-analysis was performed using OpenMetaAnalyst open-source software (http://www.cebm.brown.edu/open_meta) on eight of the nine randomised controlled trials. The study by the RMC Trial Group [18] titled “*Randomised controlled trial of methotrexate for chronic inflammatory demyelinating polyradiculoneuropathy (RMC trial): a pilot, multicentre study”*, did not fit the inclusion criteria for meta-analysis as the outcome measure was a reduction in the weekly dose of IVIg whereas the other studies measured response to the therapies. To assess overall efficacy from all the studies, we calculated odds ratio, and used a binary random-effects model to report an overall effect, heterogeneity and p-value together with 95% CI [19]. Statistical significance was declared if the p value was <0.05. Weighting for each study was also reported (Figure 1).

### Data extraction

Titles and abstracts selected were checked by the first author who also determined which studies fit the inclusion criteria. In view of the fact that different studies used different disability scales, the primary outcome measure was defined as the proportion of patients with a clinical response during or after treatment. The strictest criteria to define improvement were used in each study.

## Results

**Figure 2:**
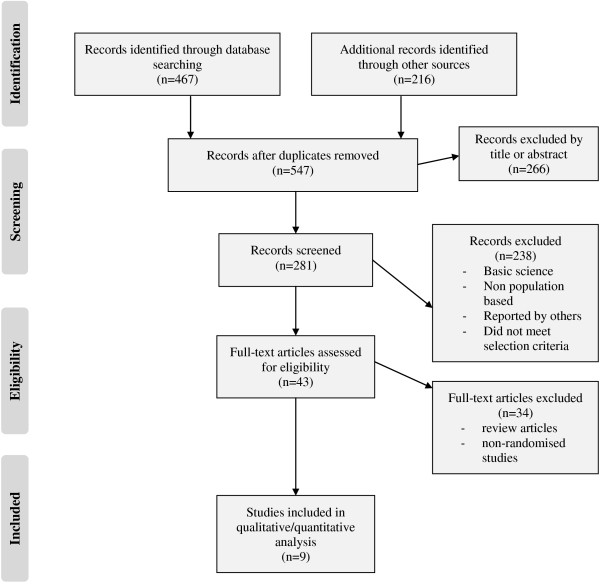
Flow diagram showing selection of articles for review.

The search terms and additional searches resulted in the identification of 540 papers from this 392 were unique (Figure 2). After reviewing the abstracts, a further 351 citations were discarded as they did not meet the inclusion criteria. After examining the full text of the remaining 41 articles a further 32 papers were excluded as they did not use widely accepted case definitions or were review articles. Nine studies met the inclusion criteria for analysis and are summarised in Table 1.

### Comparison of methotrexate with placebo

RMC Trial Group [18] compared methotrexate with placebo for the treatment of CIDP. This multicenter, randomised double blinded controlled trial compared oral methotrexate (7.5 mg per week for four weeks, 10 mg for the next four weeks and finally 15 mg for the next 32 weeks) with placebo for CIDP patients who require either IVIg or corticosteroids. Fifty nine patients out of 60 completed the trial with the primary outcome being greater than 20% reduction in weekly dose of IVIg or corticosteroids. Fourteen out of 27 (51.9%) treated with methotrexate and 14 out of 32 (43.8%) treated with the placebo exhibited a greater than 20% reduction in the mean weekly does of corticosteroids or IVIg (OR 1.38, 95% CI 0.5-3.87) (Table 1). There were no serious treatment related effects in either group to methotrexate. Oral methotrexate did not show any significant benefit, however limitations in study design and high response rate in the placebo group may have been due to a higher than necessary dose of IVIg or corticosteroids that had been administered prior to the starting the study.

### Interferon versus placebo

A clinical trial comparing interferon (IFNβ 1α) with placebo followed by IVIg was undertaken by Hughes et al.[20]. Forty five patients received either IFNβ 1α (30 μg or 60 μg intramuscular) once or twice a week plus placebo and 22 patients received placebo twice per week. Participants also received their stable IVIg regimen (2 to 5 doses per week, n = 31 received ≤ 0.95 g/kg IVIg and n = 32 received >0.95 g/kg IVIg) from week 1 through to week 16, and subjects who deteriorated post week 16 were re-administered IVIg until stable. The primary outcome was the total IVIg administered from week 16 to week 32, and secondary outcome was time to disease progression. There was no difference in the total IVIg administered after week 16 in both groups (p = 0.75), however the authors suggested IFNβ 1α reduced the total dose of IVIg in patients who required higher dose (>0.95 g/kg/month). The secondary outcome of a ≥ 2 point decrease in medical research council (MRC) sum score [21] and a 1 point increase in the overall disability status scale (ODSS), was not significant between the treatment and placebo groups (p = 0.67). Twelve out of 35 (34.3%) subjects given IFNβ 1α and 9 out of 19 (47.4%) in the placebo group exhibited disease progression from the time of IVIg withdrawal at week sixteen (OR 0.58, 95% CI 0.19-1.81, p = 0.67) (Figure 1). Adverse effects were observed more commonly in the IFNβ 1α arm, which included flu-like symptoms, headache and fatigue. The dropout rate (>20%) resulted in the study being underpowered for the detection of smaller clinically meaningful differences, and the studies were subsequently classified as a class II. There was no effect on the primary and secondary outcome measures on the four dose regimes of IFNβ 1α therapy combined with IVIg.

### Excorim staphylococcal protein immunoadsorption versus IVIg

A pilot study commenced at the Toronto General Hospital from 2003 to 2004 to evaluate the efficacy and safety of immunoglobulin removal by Excorim Immunoadsorption (IA) [22]. Twenty treatment naïve (other than prednisone) subjects with probable CIDP were randomly assigned to IA or IVIg (1 g/kg/day × 2 days). The drop-out rate over the duration of the trial was 35% due to illness and two deaths were reported, however they were unrelated to the treatment. Patients assigned to IA received three treatments over a 7 day period, with a total of 3 plasma volumes processed per treatment. Outcome measures included; nerve conduction parameters, grip strength and the Toronto Clinical Neuropathy Score (TCNS) [23] measured post initiation of treatment at 2 and 6 months. Two months after commencement of the study, 4 out 5 (80%) treated with IA and 4 out of 8 (50%) on the IVIg arm responded well to the treatment (OR 4.0, 95% CI 0.30-53.47) (Table 1 and Figure 1). At 6 months after treatment commencement 100% (4/4) of the IA arm and 50% (3/6) of the IVIg arm continued to exhibit substantial clinical response (p = 0.2). Both treatments were well tolerated, with minimal adverse effects. The study demonstrated the efficacy and safety of IA, however it was not sufficiently powered to detect significant difference between the two treatments. An appropriately powered randomised double-blind controlled clinical trial with stratification for disease duration would be required to determine the usefulness of IA therapy.

### Efficacy of IVIg therapy for CIDP

Three randomised controlled clinical trials studying the efficacy and safety of IVIg were identified and included in the review (Table 1). Leger et al. [24] undertook a double-blind, placebo controlled crossover study of nineteen subjects with demyelinating neuropathy and persistent nerve conduction block. Participants were randomly assigned to receive either IVIg (500 mg/kg/day × 5 days) or placebo (1% human albumin) once a month for 3 months. After 3 months participants had a double blind clinical assessment. Responders remained on the same treatment and non-responders switched to the alternative study drug for the remaining three months. The outcome measures were MRC score, nerve conduction and self-evaluation parameters. Follow up evaluation was performed at 4 and 7 months. Eighteen of the 20 subjects were eligible for analysis, 9 in each arm of the study. At four months 77.8% (7/9) of participants who received IVIg first and 22.2% (2/9) who received the placebo responded well to the self-evaluation parameters (OR 12.25, 95% CI 1.33-113.06, p = 0.03). No serious treatment related effects were noted in this study. Although the trial was small and not powered significantly, it did show IVIg was effective treating the symptoms of in CIDP.

Vermeulen and colleagues [25] undertook a double-blind placebo controlled multicentered trial investigating the efficacy of IVIg. Participants were randomised to receive either IVIg (n = 15) or placebo (n = 13) (Table 1). The primary outcome measure was a one point decease on the modified Rankin scale [26]. Weakness of three arm and three leg muscles on both sides of the body were also assessed using the MRC sixty point scales. The assessments were done at day 1, then again between day 16 and 21 and also after the completion of the trial. Four of fifteen (26.7%) and 3 of 13 (23.6%) subjects who received IVIg and placebo respectively improved by one point on the Rankin scale (OR 1.21, 95% CI 0.21-6.80). No significant difference was observed between the treatment and placebo groups possibly due to patient selection, treatment allocation and type II error may be a consideration due to the small sample size. There may be other factors in the placebo (20% albumin solution) that may have contributed to the clinical response seen in the placebo group, such as immunoglobulins other than IgG and proteins including albumin. It was concluded that better patient selection, selection of an appropriate placebo and larger participant number to statically power the study would be required. Further investigation would also be warranted to identify factors within the albumin fraction that may be beneficial to CIDP patients. The study did not mention adverse treatment related effects. Latov et al. [27] undertook a study investigating the timing, course and clinical characteristics of IVIg treatment for CIDP. One hundred and seventeen subjects with CIDP were randomly assigned to IVIg (Gamunex, n = 59) or placebo (0.1% albumin, n = 58) [27]. Participants receiving the IVIg, were initially administered a loading dose of 2 g/kg IVIg for 2 to 4 days followed by a maintenance dose of 1 g/kg IVIg /3 weeks for a maximum of 24 weeks. The main outcome measure was an improvement of at least one point on the INCAT disability score [28] at week six and maintained through to week twenty four. Fifty four percent (32/59) treated with IVIg were clinical responders versus 20.7% (12/58) of subjects who received the placebo (OR 4.54, 95% CI 2.01-10.28, p < 0.001).

These observations suggest that a loading dose followed by three weekly maintenance doses of IVIg may be required to achieve a maximum therapeutic response. No serious adverse effects were reported. No bias was detected in this study.

### Comparison of two different preparations of IVIg

The primary objective of a study reported by Kuitwaard et al. [29] was to compare the efficacy and safety of two different brands of IVIg for the treatment of CIDP. Twenty seven subjects were randomised to receive either the standard 5% IVIg (Gammagard S/D freeze dried IVIg, n = 13) or the new 10% liquid IVIg (Kiovig, n = 14). The primary outcome measure was a change in overall disability sum score (ODSS), and the secondary outcome measure was the MRC sum score. There was no significant difference for all measures of outcome between the two treatments. ODSS difference from analysis of covariance with adjustment for baseline values was 0.004 (Gammagard minus Kiovig) (95% CI −0.4-0.4 p = 0.98) and MRC sum score was −0.58 (95% CI −1.9-0.7 p = 0.37). The number of subjects who reported adverse effects was similar in groups, 71% in the Gammagard group and 69.2% in the Kiovig group (p = 0.86, OR 1.11, 95% CI 0.21-5.80). This study demonstrated similar efficacy and adverse effects between Gammagard and Kiovig. There was no bias detected in this study however, it was not possible to blind the nursing staff as dosages of the two IVIg preparations (Gammagard 5% IVIg and Kiovig 10% IVIg) had to be the same.

### Comparison of Rituximab with placebo

Rituximab is a humanised murine monoclonal antibody directed against CD20, a cell surface protein found on B lymphocytes the precursors of antibody producing plasma cells, thus depleting circulatory B cells. The immunotherapy drug has showed promising results in treating autoimmune diseases and has been approved for the treatment of rheumatoid arthritis [30]. A double-blind placebo controlled trial was conducted to determine the efficacy of rituximab in patients with demyelinating neuropathy [31]. Twenty six subjects with CIDP were randomised to 375 mg/m^2^ of rituximab (n = 13) or placebo (n = 13) (Table 1). Rituximab was administered in four, weekly cycles intravenously and the placebo consisted of an isotonic saline solution. The primary outcome measure was an improvement of ≥ 1 INCAT disability score at baseline and 8 months. Intention to treat analysis was not significant (p = 0.96) due to one subject treated with rituximab had a normal (0) INCAT disability score at the beginning of the trial. When participants were removed from the analysis because they did not improve with the study period the remaining data showed a significant improvement over the placebo (p = 0.036). Four out of thirteen (30%) of subjects treated with rituximab improved by ≥ 1 INCAT disability score compared to 0% in the placebo group (OR 12.79, 95% CI 0.61-266.66). No significant changes in the MRC score or nerve conduction studies were reported in the rituximab group. The most common side effects reported in the rituximab treated subjects were mild temperature increases and chills. Although the sample size was small, rituximab improved the clinical response in patients with treatment resistant demyelinating neuropathy.

### Pulsed dexamethasone versus short term standard prednisolone

A multicentered retrospective randomised controlled trial compared pulsed dexamethasone prednisolone to treat CIDP [32]. Forty newly diagnosed subjects with CIDP were randomised to receive pulsed courses of oral 40 mg per day dexamethasone for six months (n = 24) or 60 mg prednisolone daily for eight months (n = 16) (Table 1). One participant from the prednisolone arm was lost during follow-up. Based on improvement of the INCAT disability scale and RMI [26,33] the treating neurologists were asked to score treatment effect, remission, stable disease and non-responders. Ten of 24 (41.7%) subjects treated with pulsed dexamethasone and 6 of 16 (37.5%) on daily prednisolone were in remission at follow-up (OR 1.19, 95% CI 0.33-4.36). The median interval from the beginning of the remission to relapse was 11 months for prednisolone and 17.5 months for pulsed dexamethasone. Adverse treatment related events were low in both groups. There was no difference between treating subjects with either pulsed dexamethasone or standard daily prednisolone, however long-term remission was possible in 25% of sufferers with CIDP after only one or two courses of monthly dexamethasone compared with eight months of daily prednisolone. The authors failed to analyse the treatment related responses for statistical significance due to heterogeneity. At follow-up many of the non-responders were determined to have been misdiagnosed. Randomisation was achieved with a random number generator, however the study failed to conceal allocation and there was an absence of blinding [34].

## Discussion

The purpose of this systematic review was to evaluate recent randomised clinical trials to determine the efficacy and safety of current therapies in the recovery of CIDP. Overall all treatment modalities for CIDP favoured a positive response (OR 2.10, 95% CI 0.96-4.61, p = 0.05) (Figure 1). Two important observations arose from this review; firstly, there is a need to use better objective scales to measure disability and assess long-term outcomes and responses to treatments. Secondly there is a need to be able to identify and omit subjects with stable or inactive disease, as they would be naturally less likely to respond to new or novel treatments. Recent research illustrated that up to 40% of subjects with CIDP were in remission or cured, but included in clinical trials, however CIDP patients can exhibit spontaneously improvement. This may explain why there was a high placebo response rate observed in some clinical trials [15,27]. Using a reliable scale such as CIDP Disease Activity Status (CDAS) in patient recruitment for clinical trials would provide more meaningful data with fewer patients. Constructing better powered larger studies would also assist in demonstrating potentially important small differences and in data interpretation. The data from the systematic review identified, that IVIg appeared to provide an effective treatment option for subjects with active CIDP, particularly evident in the studies by Zinman et al. [22], Latov et al. [27] and Leger et al. [24]. Further studies are necessary to investigate plasma factors other than immunoglobulins in IVIg, which may result in an increase in clinical response rates in trials using albumin as placebo. Replacing albumin with isotonic saline would be a better choice for the placebo. As discussed previously, this phenomenon was seen in an IVIg versus placebo trial undertaken by Vermeulen et al. [25], however the high clinical response rate observed in the control arm may also be due to patients being incorrectly diagnosed or possibly spontaneous recovery from the disorder. Rituximab showed promising results [31], however larger randomised clinical trials with long-term follow up would be required. Heterogeneity was moderate between the eight randomised controlled studies included (Tau^2^ = 0.58, Q = 14.05, I^2^ = 50% and p = 0.05) and due to the variability of the disease the binary random effect model was used in this study. This was to be expected as the treatments varied considerably and it was not the purpose of this review to combine studies. The systematic review did not include plasma exchange therapy, however two double crossover trials identified did not fit inclusion criteria and had been previously reported [35]. The two clinical trials using plasma exchange for the treatment of CIDP demonstrated a significantly better outcome in the treatment groups [36,37]. Due to the rarity and numerous variants of CIDP, limited large clinical trials have been undertaken. Clinical trials with similar autoimmune inflammatory diseases such as Guillain–Barré syndrome, multiple sclerosis and Crohn's disease may also provide some guidance in treatment options for those suffering from CIDP. Two compounds, Kv1.3 and KCa3.1 (voltage-gated potassium channel and calcium activated potassium channel inhibitors respectively) have shown promising results in animal studies for the treatment of autoimmune diseases such as multiple sclerosis, psoriasis and type-one diabetes may also benefit subjects with CIDP [38,39]. Currently a clinical trial is underway to observe whether alpha lipoic acid, an antioxidant with anti-inflammatory properties may prove effective to treat CIDP symptoms (ClinicalTrials.gov Identifier: NCT00962429). Hematopoietic stem cell transplantation for treating CIDP is also showing some promise [40], with a clinical trial actively recruiting participants (ClinicalTrials.gov Identifier: NCT00278629). With the development of antigen arrays, customised DNA vaccines may have potential to cure CIDP and other autoimmune disorders by tolerizing against an aberrant immune response observed in subjects with CIDP.

## Conclusion

This systematic review demonstrated that IVIg and corticosteroids still provide the most effective first line treatment options for patients with active CIDP. The review also revealed there is a pressing need for further basic research into the pathogenesis of CIDP to ultimately develop new therapies for more effective treatment. Larger randomised controlled studies are required to define the validity and efficacy of treatments such as stem cell transplantation and immune-modulating agents. Better definition of CIDP is also required due to wide spectrum and the variability of clinical presentations of CIDP, together with the use of a valid disability scale such as CDAS, to ultimately lead to better subject selection for long-term studies of CIDP therapies.

## Abbreviations

IVIg: Intravenous immunoglobulin; CIDP: Chronic inflammatory demyelinating polyradiculoneuropathy; MAG: Anti-myelin associated glycoprotein; PE: Plasma exchange; MRC: Medical research council; ODSS: Overall disability sum score; CDAS: CIDP Disease activity status.

## Competing interests

The authors declare that they have no conflicting interests.

## Authors’ contributions

RB conceived the study and design, all acquisition of data, analysis and interpretation of data and drafting the manuscript. JW & BC critically revised the manuscript. All authors read and approved the final manuscript.

## Pre-publication history

The pre-publication history for this paper can be accessed here:

http://www.biomedcentral.com/1471-2377/14/26/prepub
